# Localization of the immunodominant region on human thyroid peroxidase in autoimmune thyroid diseases: an update

**DOI:** 10.1186/1740-2557-2-2

**Published:** 2005-03-15

**Authors:** Damien Bresson, Sandra A Rebuffat, Sylvie Péraldi-Roux

**Affiliations:** 1 CNRS UMR 5160, Centre de Pharmacologie et Biotechnologie pour la Santé, Faculté de Pharmacie, 15 avenue Charles Flahault, BP 14491, 34093 Montpellier Cedex 5, France; 2 La Jolla Institute for Allergy and Immunology, Department of Developmental Immunology-3, 10355 Science Center Drive, San Diego, CA 92121, USA

**Keywords:** Autoimmunity, Thyroid Peroxidase (TPO), Autoantibody (aAb), Immunodominant region (IDR), Autoimmune Thyroid Diseases (AITD).

## Abstract

Recent studies in the field of autoimmune thyroid diseases have largely focused on the delineation of B-cell auto-epitopes recognized by the main autoantigens to improve our understanding of how these molecules are seen by the immune system. Among these autoantigens which are targeted by autoantibodies during the development of autoimmune thyroid diseases, thyroid peroxidase is a major player. Indeed, high amounts of anti-thyroid peroxidase autoantibodies are found in the sera of patients suffering from Graves' disease and Hashimoto's thyroiditis, respectively hyper and hypothyroidism. Since anti-thyroid peroxidase autoantibodies from patients'sera mainly recognize a discontinuous immunodominant region on thyroid peroxidase and due to the complexity of the three dimensional structure of human thyroid peroxidase, numerous investigations have been necessary to closely localize this immunodominant region. The aim of the present review is to summarize the current knowledge regarding the localization of the immunodominant region recognized by human thyroid peroxidase-specific autoantibodies generated during the development of autoimmune thyroid diseases.

## 1. Introduction

Autoimmune Thyroid Diseases (AITD) are one of the most frequent organ-specific autoimmune diseases affecting more than 3% of the total population worldwide. The clinical spectrum can be divided into two major subtypes, (i) glandular hyperfunction characterized by Graves' disease, and (ii) glandular hypofunction as Hashimoto's thyroiditis. In both diseases, there is a breakdown in tolerance and the generation of an immunoglobulin G response directed against thyrotropin receptor (predominantly in Graves' disease [1,2]), thyroglobulin, and thyroid peroxidase (TPO). Autoantibodies (aAbs) against TPO, a common denominator of AITD, are present in 90% of Hashimoto's thyroiditis and 75% of Graves' disease patients' sera [3]. Since anti-TPO aAbs are present at high concentration in patients' sera [4,5], they are invaluable makers of the thyroid autoimmune response and thus extensively used to diagnose such pathologies. *In vitro *and *in vivo *cytotoxic effector functions have also been described such as C3 complement activation [6-8] and antibody-dependent cell cytotoxicity [9-12] leading to the maintenance and amplification of thyroid cell destruction in Hashimoto's disease. Moreover, anti-TPO aAbs are likely to play a more important role in presenting TPO to T cells [5,13,14]. To understand the role of anti-TPO aAbs in the pathogenesis of AITD and to shed new light on how the TPO molecule is seen by the immune system, the delineation of anti-TPO B-cell epitopes has been the goal of several studies during the last decades. These findings enabled a better localization of the discontinuous immunodominant region (IDR) with the description of several amino acid residues taking part in this highly complex structure. All together, these data could be a great interest to rationally design competitors (such as peptides) which could be used in combination with other immunotherapies such as systemic antibody treatment, antigen-specific immunization or others generating antigen-specific regulatory T cells capable to block at least for a period of time, an ongoing autoimmune process and may synergize to delay hypothyroiditis.

## 2. TPO : structure and function

**Figure 1:**
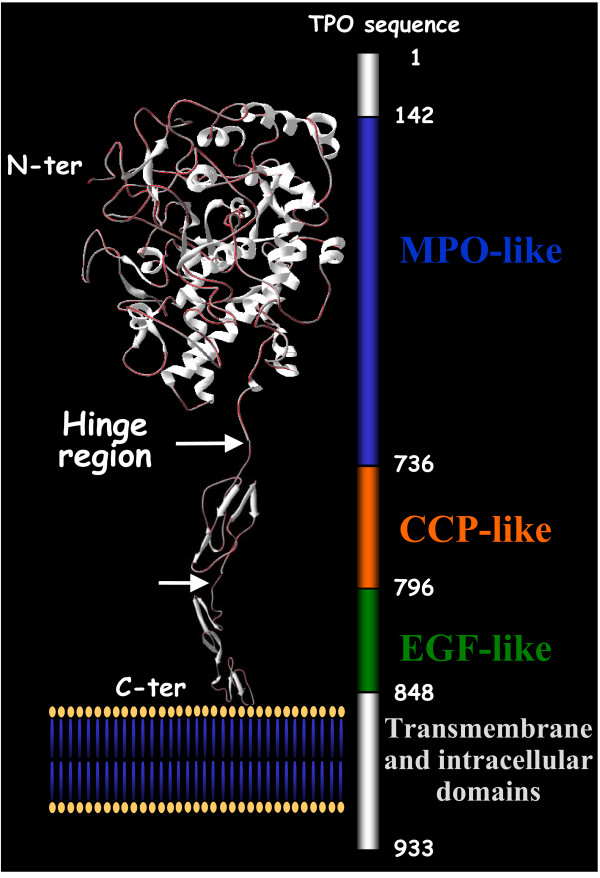
**Ribbon diagram of the structure of human TPO. **(Reproduced with permission 14). The MPO- (residues 142–733), CCP- (residues 739–795) and EGF-like (residues 796–841) domains are indicated with a black arrow. This representation corresponds to the juxtaposition of the three-dimensional model of each domain.

TPO is a membrane-bound, glycosylated hemoprotein that plays a key role in thyroid hormone synthesis by catalyzing both the iodination of thyroglobulin and the coupling of some of the iodotyrosyl residues to generate the thyroid hormones T3 and T4. Human TPO (hTPO) is a 933 amino acid type I integral apical membrane protein that contains a large extra-cytoplasmic domain orientated toward the follicular lumen, a short membrane-spanning region, and a 61 amino acids cytoplasmic tail. The extra-cellular region consists of 848 amino acids and five potential glycosylation sites. Alignment studies and structural homologies have shown that hTPO is formed by three distinct domains: a myeloperoxidase (MPO)-like, a complement control protein (CCP)-like, and an epidermal growth factor (EGF)-like domains, from the N- to the C-terminal extremities [15] (Figure 1). The high homology between the MPO and TPO molecules has allowed the prediction of the secondary structure and domain organization of TPO [16] which is composed mainly of alpha-helical structure with relatively little beta-sheet structures. More recently, the structure of each domain has been partially elucidated by three-dimensional modeling [17,18]. The three-dimensional structure of TPO, however, remains unknown, even though low resolution crystals have been obtained [19,20]. TPO is known to exist as multiple species and migrates on SDS PAGE analysis as a closely spaced doublet, which derives from endogenous proteolysis [21,22]. The multiplicity of N-terminal truncated forms of TPO contributing to the diversity of the polypeptide chain, and the flexibility observed for the hinge regions leading to an instable form of TPO, have made difficult to obtain high resolution diffracting crystals for three dimensional structural analysis. Probably, the only way to solve the three-dimensional structure of TPO will be to "freeze" the molecule in a stable conformation by crystallizing a complex TPO/anti-TPO Fab. This strategy would be also greatly informative to define all residues involved in the interaction. Nevertheless, because of the difficulty in obtaining diffracting crystals, other strategies, such as competition between anti-TPO aAbs, site-directed mutagenesis or construction of chimera, have been used to delineate B-cell autoepitopes on the TPO molecule [17,23].

## 3. Anti-TPO autoantibodies and immunodominant region

Anti-TPO aAbs are specific serological markers for diagnosing AITD. Anti-TPO aAbs are produced by B lymphocytes via a T cell-dependent mechanism. Since several years, it is well known that anti-TPO aAbs recognize conformational epitopes that are highly dependent on the three-dimensional structure of the TPO molecule [2]. These TPO aAbs are known to be restricted to two immunodominant regions (IDR) containing different but adjacent surface epitope. In 1989, Ruf and Carayon's group [24] pioneered in the localization on antigenic domains on hTPO. Using a panel of monoclonal antibodies (mAbs) with different affinities and epitopic specificities for hTPO, they studied the distribution of epitopes on the surface of the hTPO molecule. These mAbs were found to interact with four domains (termed A to D) on native TPO. But, in competitive experiments with sera from patients suffering from AITD, only the binding of mAbs interacting with the two major antigenic domains (named A and B) was highly inhibited by anti-TPO aAbs from patients' sera, demonstrating that these two regions form the IDR on the hTPO.

During the last two decades, an explosion in the development of combinatorial libraries technologies enable the production of anti-TPO human mAb fragment from several phage display antibody libraries constructed by using thyroid infiltrating B-cells from Graves' disease or Hashimoto's thyroiditis patients. Using a panel of four anti-TPO antibody fragments (Fabs), named SP1-5, WR1-7, TR1-8 and TR1-9, Chazenbalk and co-workers [25] showed that these four recombinant human Fabs inhibit more than 90% of anti-TPO aAbs present in patients' sera affected by AITD. Thus, they have been concluded that these four Fabs recognize epitopes that delineate the IDR of hTPO. Interestingly, the IDR was found to be composed by two overlapping regions (named A and B domains) as previously described with the mouse mAbs (but the domains were described with an inverted nomenclature) [25-27]. Our group also contributed to increase the number and diversity of human monoclonal anti-TPO aAbs using the phage display technology. Forty-four human anti-TPO aAbs were selected from one "in cell" library and three random combinatorial libraries constructed from different B-cell subsets extracted from thyroid biopsies from Graves' disease patients [28,29]. These recombinant human anti-TPO aAbs, mimicking the human repertoire, strongly displace the binding of anti-TPO aAbs present in patients' sera (80% inhibition) to hTPO, and predominantly recognize the IDR.

Competitive experiments between human anti-TPO aAbs from patients' sera and anti-TPO aAbs (human Fabs or mouse mAbs), were used to draw contours of the IDR/A and IDR/B on the TPO molecule. However competition studies do not allow the precise localization of the IDR as well as the identification of the amino acid residues involved in the interaction. Consequently, other approaches were investigated with the aim of (i) defining the contribution of each domains (A and B) in the IDR, (ii) identifying key residues involved in the IDR/A and IDR/B-specific human anti-TPO aAbs epitopes, and (iii) understanding the relationship that could exist between both domains.

## 4. Contribution of the MPO-like and CCP-like domains in the IDR

The MPO-like domain has been clearly found to contribute in the folding of the IDR (IDR/A and IDR/B). It is important to note that the A domain defined by the murine mAbs corresponds to the B domain defined by the human Fabs and *vice versa*. Since we have used human anti-TPO aAb in our experiments as did McLachlan and Rapoport's group [30], we use here the nomenclature of the IDR/A and B defined by this group. Banga and Gardas' groups chose to produce a panel of polyclonal anti-sera by immunizing rabbits with a series of overlapping or adjacent peptide sequences exposed at the surface of TPO (based upon the TPO model). By using a rabbit antiserum against the peptide P14 (aa 599–617), they successfully located the IDR/A epitope on hTPO in the MPO-like domain. Importantly, this region is included in the recombinant fragment of hTPO (aa 589–633) which was previously identified as an autoantigenic determinant [31].

**Figure 2:**
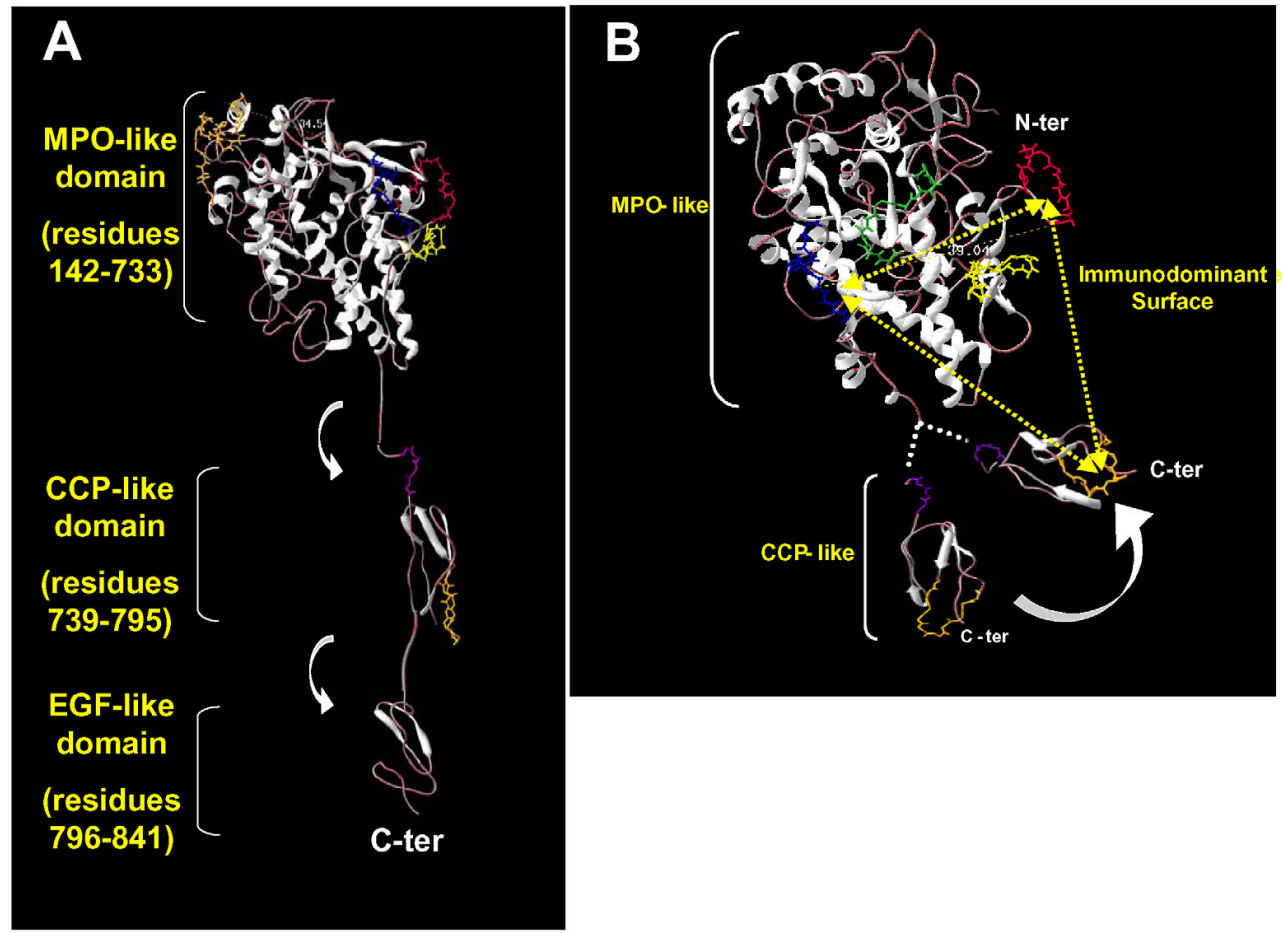
**Location of the regions participating in the IDR. **(A) Regions involved in the binding with aAbs are indicated on a three-dimensional diagram of the human TPO : positions 353–363 in yellow, 377–386 in red, 506–514 in green, 713–720 in blue, 599–617 in orange in the MPO-like domain, 737–740 in pink, and 766–775 in orange in the CCP-like domain. The flexibility of the hinge regions is represented by a white arrow. (**B**) Ribbon diagram representing the possible folding of the CCP-like domain onto the MPO-like domain. An immunodominant binding surface is virtually represented by three dotted lines and the distance (in Å) between the regions 377–387 and 713–720 is shown. The model was adjusted by using Swiss-PDB viewer 3.7b2 freeware available at .

Amongst the panel of mAbs produced by Ruf and Carayon's group [24], only mAb 47 binds to both, denatured and native TPO. This mAb recognizes a linear epitope first located between amino acid residues 713–721 [32] and later restricted to residues 713–717 [33]. Interestingly, mAb 47 was shown to strongly competes with IDR/B-specific human anti-TPO aAbs thus defining for the first time a short immunoreactive IDR/B-specific region [30,37]. Furthermore, mutations between the positions 713–717 specifically affected the binding of patients' sera to hTPO, definitively demonstrating that this region in MPO-like domain is involved in the IDR/B epitope. In the past five years, three other regions (210–225, 353–363, 549–563) [15,33,34], structuring the IDR/B have been identified in the MPO-like region (Figure 2A). Finally using a chimeric molecule between TPO and MPO molecules, the 121 amino-terminal residues of TPO were excluded from the IDR [35].

On the other hand, the participation of the CCP-like domain in the IDR was more difficult to demonstrate. In 1998, Estienne *et al*, [36] located a conformational B-cell epitope at the C-terminal end of TPO near the membrane anchorage domain of the molecule (amino acids 742–848). Since the majority of anti-TPO aAbs from Hashimoto patients' sera recognized a recombinant polypeptide encompassing this region, the authors thought that this region was part of the IDR. One year later, using reticulocyte lysate cell-free translation approach of TPO, Grennan *et al *[37] observed that truncation of TPO downstream of amino acid residue 771 had little effect on aAb recognition in all patients' sera tested, suggesting that the IDR recognized by human aAbs should lie between amino acid residues 742 and 771. This observation was in agreement with the early study from Estienne *et al*, but later, Xiong *et al *[38], using the cell-free translation approach, concluded that the region 742–771 does not contain the IDR. These contradictory data underline the difficulty of determining the contribution of the CCP-like domain in the IDR, specially when the CCP-like domain is expressed and studied outside the three-dimensional context of the hTPO (as exemplified with the *in vitro *translation experiments). To overcome such problems, and with the aim to localize the discontinuous IDR of the TPO, we have combined two technological advances (i) the selection of mimotopes by screening phage display peptides libraries on an IDR/B-specific human recombinant anti-TPO aAb (T13) mimicking aAbs from patients' sera, and (ii) the sequence alignment of the selected mimotopes on the primary sequence of hTPO [33]. We identified four distinct regions distributed between the MPO- and CCP-like domains. The sequences 353–363, 377–386, and 713–720, are located in the MPO-like domain while the last one, in position 766–775, is located in the CCP-like domain. More interestingly, mutation of the region 766–775 abrogated the binding of human anti-TPO aAbs from Hashimoto's and Graves' disease patients. In the contrary, the EGF-like domain of the TPO molecule was clearly excluded from the IDR [39], as well as the contact region between the two TPO monomers during the dimerisation of the molecule [35].

All together, these data pointed out (i) the participation of several regions in the MPO-like domain and at least one region (766–775) in the CCP-like domain for the folding of the IDR and (ii) that one major region (599–617) belongs to the IDR/A and five regions (210–225, 353–363, 549–563, 713–720, and 766–775) structure the IDR/B. This emphasizes the discontinuous nature as well as the complexity of the IDR and provides new insights into the MPO-like and CCP-like domains positioning. It is now obvious that the MPO-like and CCP-like domains have to be closed in the three-dimensional structure of hTPO to form an immunodominant binding surface recognized by the majority of anti-TPO aAbs, thus one could hypothesize that the CCP-like domain may be able to fold onto the MPO-like domain to enable the binding of a single aAb simultaneously to the MPO- and CCP-like domains (Figure 2B) [33].

## 5. Amino acid residues structuring the IDR

To improve our knowledge of IDR and to understand how the TPO is seen by the immune system during the pathogenesis of AITD, it was also important to determine the amino acid residues involved in the epitopes recognized by human anti-TPO aAbs. Among the major regions identified over time, contributing amino acid residues have been identified. By expressing recombinant hTPO mutants with single amino acid replacements, several groups participated to the characterization of critical amino acid residues. [17,34,40,41]. Although, seven key residues (R225, Y772, and K713 to D717) involved in the IDR/B-specific anti-TPO aAbs epitope were identified, only one amino acid residue (K627) has been clearly assigned to the IDR/A-specific human anti-TPO aAb epitope.

## 6. Respective positioning of IDR/A and IDR/B

**Figure 3:**
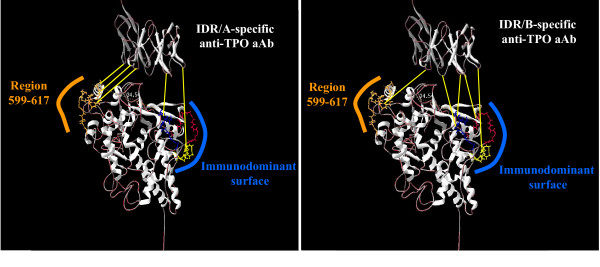
**IDR/A and IDR/B-specific anti TPO aAbs mainly recognize the same regions on hTPO: the binding strength makes the difference. **IDR/A- and IDR/B- specific epitopes (left and right panel respectively) are globally composed by the same peptidic regions on hTPO. Region 599–617 (in orange) represents the amino acid sequence predominantly recognized by IDR/A-specific anti-TPO aAbs (as illustrated by three yellow lines on the left panel). On the hand, IDR/B-specific anti-TPO aAbs bind several regions widely spread on the IDR. The immunodominant binding surface (in blue) and region 599–617, composed the IDR/B-specific epitopes as shown by the yellow lines which illustrate the contact points on hTPO. As not shown in the figure, the CCP-like domain may contribute to the immunodominant epitopes.

The two main discontinuous IDR (known as IDR/A and IDR/B) have been studied for several years to identify the TPO amino acid sequences involved in the binding of human anti-TPO aAbs. Over time, a body of evidence demonstrated that IDR/A and IDR/B, although different, overlap in part [25,27]. Thus, in spite of the growing number of data available for delimiting each domain (A and B) forming the entire IDR, it was unclear whether these domains (i) form two separate clusters recognized by either IDR/A- or IDR/B-specific human anti-TPO aAbs or (ii) are structurally similar (involving the same surface area on the TPO molecule). Using all the reference anti-TPO Abs (recombinant human Fabs, mouse mAbs and rabbit polyclonal anti-peptides), with the same approach, the relationship between IDR/A and IDR/B domains has been investigated [42]. We have demonstrated that human IDR/A and IDR/B-specific anti-TPO aAbs recognize essentially the same region on the surface of hTPO. However, IDR/A-specific aAbs use the region 599–617 as anchor point for their binding to hTPO much more forcefully (Figure 3).

Such observations emphasize, once again, the high complexity of the IDR on the TPO molecule. It has been now well demonstrated that the IDR is stretched along the MPO- and CCP-like domains of hTPO. Consequently, some regions such as the region 599–617, 713–720, and 772–775 seem to be too far, on the three dimensional model of hTPO, to compose a single epitope, albeit discontinuous. Furthermore, a rapid inspection of the three dimensional TPO model shows that region 599–617 (the main anchor point for the IDR/A-specific aAbs) is located on the opposite side of the MPO-like domain with regard to the immunodominant binding surface composed of three auto-reactive TPO peptide sequences (353–363, 713–720 or 766–775) (Figure 2B). Until now, the only three-dimensional structure of hTPO known is a computer model and the whole structure of hTPO could be more compact than the model suggested. Thus, a flexibility in the hinge region between the MPO-, CCP- and EGF-like domains would lead to the formation of a more closely folded molecule [17,33]. Furthermore, the membrane-bound TPO, at the surface of thyrocytes as well as CHO cells, exists as a disulfide-linked dimer [43]. Thus the presence of TPO dimer could favor a spatial gathering of the IDRs recognized by IDR/A and IDR/B-specific anti-TPO aAbs. Since, anti-TPO aAbs epitopes are largely restricted to one facet of the native molecule [24,25,33] this may enable the formation of a dimer by the other side, as previously modeled by homology with the crystal structure of the MPO molecule [15].

## 7. Concluding remarks

Numerous efforts have been made to precisely characterize the regions, as well as the amino acid residues forming the IDR of hTPO. This could lead to the rational design of therapeutic peptides able to modulate or block immune responses such as antigen presentation. Thus, it would be of great interest to determine *in vivo*, by using animals models for AITD, whether it will be possible to influence the diseases' course (rapidity, severity, etc...) by using these peptidic immunomodulators interacting with antigen presentation of B-cells to T-cells.

However, a major question remains: What is the clinical significance of the anti-TPO aAbs which are present in the two well characterized AITD (Graves' disease and Hashimoto's thyroiditis)? Although, there is a general consensus that one of the most important markers of thyroid autoimmunity is the production of such thyroid-specific aAbs. Their role during the pathogenesis of AITD is still controversial.

Several lines of evidence show that there is no difference in the epitope recognized by anti-TPO aAbs produced during Graves' disease, Hashimoto's thyroiditis and Euthyroiditis [44-46], and it is well-known that epitope spreading phenomenon does not occur in patients suffering from these diseases, at least for the B-cell epitopes [5]. Since, the components of the IDR/A and IDR/B of hTPO are now well characterized, it would be interesting to evaluate by longitudinal studies, whether the epitopic pattern of individual patients is changing (from an IDR/A to IDR/B pattern for example or *vice versa*) during the course of the diseases or after treatment.

## List of Abbreviations

Thyroid Peroxidase; TPO, Autoantibody; aAb, Autoantigen; aAg, Immunodominant Region ; IDR, Autoimmune Thyroid Disease; AITD, Complement Control Protein; CCP, Myeloperoxidase; MPO, Epidermal Growth Factor; EGF.

## Competing interests

The author(s) declare that they have no competing interests.
